# Clinical Management of Sarcopenia: Secondary Publication of Geriatrics ＆ Gerontology International 2018;18 S1:1-44

**DOI:** 10.31662/jmaj.2019-0032

**Published:** 2020-03-06

**Authors:** Hidenori Arai

**Affiliations:** 1 National Center for Geriatrics and Gerontology, Obu, Japan

**Keywords:** sarcopenia, frailty, muscle, protein, exercise

## Abstract

In aging societies, sarcopenia is considered to be a significant threat for the elderly and for people with multimorbidities. Although several diagnostic algorithms are currently available, no guidelines are so far available for the prevention and intervention of sarcopenia. Therefore, we decided to publish clinical sarcopenia guidelines by collaboration with the Japanese Association on Sarcopenia and Frailty, the Japan Geriatrics Society, and the National Center for Geriatrics and Gerontology to provide tools for clinical practice. We published a Japanese version in December 2017 and an English version in May 2018. This article is a summary of these clinical sarcopenia guidelines. As the disease code of sarcopenia is available in Japan, these guidelines would be useful for many healthcare professionals and can be used for the prevention of disabilities in the elderly.

## Introduction

Nearly 30 years have passed since the term “sarcopenia” was coined by Rosenberg to describe a syndrome characterized by age-related loss of skeletal muscle mass ^(1)^. Extensive research has been carried out on the pathogenesis and epidemiology of sarcopenia to establish the concept of sarcopenia. The first operational definition for sarcopenia diagnosis was proposed by the European Working Group on Sarcopenia in Older People (EWGSOP) in 2010 ^(2)^. They described that sarcopenia not only is a health threat in the elderly but also affects the prognosis of older people suffering from various diseases. After the publication of the EWGSOP definition, several groups proposed an algorithm of sarcopenia diagnosis and most of them adopted muscle mass measurement and muscle strength/physical performance measurement for diagnosis ^(3)^. Recently, sarcopenia has been recognized as an independent disease and was assigned the ICD-10 code M62.84 on October 1, 2016. In order to raise the awareness of sarcopenia in clinical practice, we decided to establish clinical guidelines for sarcopenia, which we will introduce in this article.

## Diagnosis of Sarcopenia

**Figure 1.**
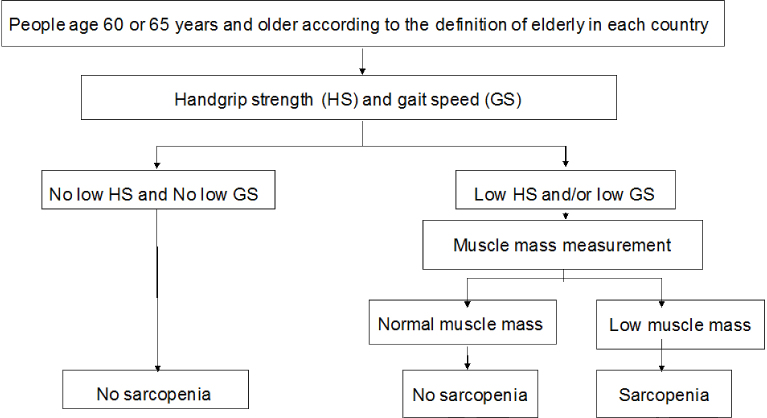
Diagnostic criteria by the AWGS ^(4)^. Quated from Chen LK, Liu LK, Woo J, et al. Sarcopenia in Asia: consensus report of the Asian Working Group for Sarcopenia. J Am Med Dir Assoc. 2014;15(2):95-101 (4) This figure is used under the permission.

In these guidelines, we recommend the use of the diagnostic criteria established by the Asian Working Group for Sarcopenia (AWGS) (Figure 1) ^(4)^. First, the grip strength and usual gait speed should be measured. The proposed cutoff for grip strength is <26 kg in men and <18 kg in women. The cutoff of the usual gait speed is ≤0.8 m/s. If a low grip strength or slow gait speed is present, the next step is to measure muscle mass. In order to evaluate the appendicular skeletal muscle mass, the appendicular lean body mass or limb muscle mass should be measured using dual-energy X-ray absorptiometry (DXA) or bioimpedance analysis (BIA) and then divided by the square of the body height. However, we should be careful because the value of the appendicular skeletal muscle mass measured using the BIA method depends on the device and its output software ^(5)^. Measurements using DXA also exhibit a similar problem. Measuring the calf circumference can substitute the appendicular skeletal muscle mass measurement, and the cutoff can be <34 cm in men and <33 cm in women ^(6)^. A significant number of researchers have been working on appendicular muscle mass measurement using ultrasonography. Recently, Nijholt et al. performed a systematic review on appendicular muscle mass measurement using ultrasonography ^(7)^. Although they found that the reliability and validity of ultrasound in quantifying muscles in the elderly are fairly good, most of the studies were cross-sectional in nature; therefore, outcome-based studies are necessary to provide a cutoff value for low appendicular muscle mass.

In our guidelines, sarcopenia was classified into primary and secondary ^(3)^. If the cause of muscle loss is not clear except for age, it is defined as primary sarcopenia, whereas it is regarded as secondary sarcopenia when one or more causes are obvious. The AWGS criteria in our guidelines can be used for both primary and secondary sarcopenia. However, for primary sarcopenia, diagnosis can be made only for persons aged 65 and above, whereas there is no age limitation for secondary sarcopenia.

Sarcopenic obesity is a related state characterized by simultaneous presence of sarcopenia and increased fat mass; however, there is no established definition at the moment ^(3)^. In previous studies concerned with sarcopenic obesity, the body mass index (BMI), body fat percentage, and waist circumference were used for the assessment of obesity ^(8)^. However, we do not think that the BMI can be used to assess obesity in cases of sarcopenic obesity, because any increase in body weight due to greater fat mass accompanies a decrease in body weight due to loss of skeletal muscle mass. Moreover, the definition of obesity differs between western and Asian ethnicities. As a result, the proposed definitions for sarcopenic obesity vary. In our personal opinion, sarcopenic obesity should be defined on the basis of the combination of low grip strength and high fat mass defined by increased body fat percentage. Future discussions are necessary to define sarcopenic obesity in Asian ethnicities.

## Epidemiology of Sarcopenia

Needless to say, the prevalence of sarcopenia depends on the diagnostic algorithm of sarcopenia. For example, the prevalence of sarcopenia according to the EWGSOP definition was 1–29%. The prevalence of sarcopenia was 11–24% in reports from Japan according to the EWGSOP and AWGS definitions. In a large-scale survey targeting Japanese subjects, the prevalence of sarcopenia was found to be in the range 7.5–8.2% ^(9)^. Additionally, the prevalence of sarcopenia depends on the characteristics of the study population. Among senior citizens residing in care facilities, the prevalence is higher than among community-dwelling older people. When this population also includes disabled individuals or those hospitalized to undergo rehabilitative care, the prevalence rate even becomes higher ^(10)^. Thus, specifying the prevalence of sarcopenia is difficult because determining the presence of this condition depends on the definition applied and the individual attributes of the study subjects. However, when reviewing the results of large-scale studies involving 1,000 or more subjects, the prevalence is usually in the range 6–12% ^(9)^.

Although the prevalence of sarcopenia is approximately 10% in the general population, the prevalence of sarcopenia in patients with stable chronic obstructive pulmonary disease was 14.5% when the EWGSOP criteria were applied, and no sex-based differences in prevalence were found according to a British study ^(11)^. Although no reports were published concerning EWGSOP- or AWGS-defined sarcopenia in patients with cancer, the prevalence of low skeletal muscle mass has been reported to be much higher among patients with gastric/esophageal cancer, colorectal cancer, hepatocellular carcinoma, pancreatic cancer, and renal cancer ^(9)^. In addition, based on a recent meta-analysis, solid cancers complicated with reduced skeletal muscle mass are associated with a poor prognosis and greater skeletal muscle mass is associated with better survival ^(12)^. The prevalence of sarcopenia in patients with predialysis-stage chronic kidney disease (CKD) (G3–G5) has been reported to be 5.9% ^(13)^ and 14% ^(14)^. Based on a study on South Korean patients who had reduced skeletal muscle mass (presarcopenia), the prevalence of sarcopenia was 4.3% in healthy subjects and in subjects with CKD Stage 1, 6.3% in subjects at Stage 2, and 15.4% in subjects at Stages 3–5 ^(15)^. The proportion of patients with low skeletal muscle mass was higher as CKD progressed to more severe stages. Based on the results of a survey conducted in the USA, the risk of presarcopenia is 2.58 times higher in patients with CKD Stage 4 compared to subjects without CKD ^(16)^. Although the prevalence of sarcopenia in patients in the dialysis stage of CKD has been reported to be in the range 12.7–33.7%, this wide variation can be explained by the differences in the mean age of the study subjects and in the diagnostic criteria for sarcopenia ^(17)^.

Many reports described the relationship between osteoporosis and sarcopenia. Complication by sarcopenia in osteoporosis causes falls because of the decrease in muscle mass and muscle strength, leading to further loss of bone mineral density and bone strength, resulting in osteoporotic fragility fractures ^(18)^. Conversely, the presence of osteoporosis significantly increases the risk of developing sarcopenia in the future ^(19)^. In the European Male Ageing Study, the presence of sarcopenia was found to be associated with reduced bone mineral density and osteoporosis ^(20)^. Yoshimura et al. also examined the relationship between osteoporosis (WHO criteria) and sarcopenia (AWGS criteria) in 1,099 community-dwelling subjects and found that the prevalence of osteoporosis was 24.9% ^(19)^ and that 18.9% of people with osteoporosis had sarcopenia, whereas the prevalence of sarcopenia was 8.2% and that of osteoporosis was 57.3% in sarcopenic people. The prevalence of sarcopenia was also higher among patients residing in care facilities, and the rate of complication by osteoporosis was also high ^(21)^. Although the risk of complication by sarcopenia increases in malnourished patients, several reports have discussed the roles of proteins, amino acids, and antioxidants in the pathogenesis of this condition ^(22)^. The prevalence and severity of sarcopenia are higher in cases of cachexia, and it has been reported in various studies that sarcopenia occurs in people with physical frailty ^(23)^.

## Outcome of Sarcopenia

Based on the results of an observational study in East Asia, the risks of cardiovascular death and all-cause mortality were higher in older adults with sarcopenia, particularly in those with sarcopenic obesity ^(9)^. Additionally, in was found in a study that those with sarcopenic obesity are more likely to develop dyslipidemia compared to nonobese subjects with sarcopenia ^(24)^. It has also been reported that sarcopenia is an important prognostic factor for mortality ^(25)^. In the analysis of the third National Health and Nutrition Examination Survey (NHANES III) conducted in the USA, sarcopenia was shown to be involved in glucose metabolism independently of obesity, and it was also shown that this trend was stronger among people below 60 years of age ^(26)^. It was also suggested that decreases in skeletal muscle mass are a predictor of diabetes. The relationships among sarcopenia, sarcopenic obesity, and metabolic syndrome have been shown in several cohorts ^(27), (28)^. The presence of sarcopenia is also associated with a higher risk of bone fractures in elderly men ^(29)^. Among older individuals undergoing emergency surgery, the risk of death was higher for those who had sarcopenia compared to those who did not ^(30)^. Sarcopenia has been reported to be a predictor of complications or death in patients with liver cirrhosis and hepatocellular carcinoma, as well as those following hepatectomy ^(31), (32)^. The presence of sarcopenic obesity can also increase the risk of infections after cardiac surgery ^(33)^. A study using the data of dialysis patients revealed that reduced muscle strength was associated with protein-energy wasting, decreased physical activity, inflammation, and mortality risk ^(34)^. Thus, sarcopenia is an important predictor of adverse health outcomes in critically ill patients; therefore, assessment of sarcopenia should be conducted in these patients.

## Prevention of Sarcopenia

Appropriate diet and exercise are the key for the prevention of sarcopenia. Considering that the decline of skeletal muscle starts in the thirties, it is better to prevent sarcopenia from at least the middle age. In this section, the guideline committee members systematically reviewed several articles to address the effects of diet, exercise, and other types of intervention to prevent sarcopenia ^(35)^.

In terms of diet intervention for the general public, 104 female older adults underwent dietary interventions involving increased protein intake. The subjects were assigned to either a normal-protein-intake group (0.8 g/kg/day) or a high-protein-intake group (1.2 g/kg/day), and the dietary intervention was performed with a calorie-restricted diet (20–25 kcal/kg/day) for three months ^(36)^. As a result, the muscle mass index was significantly low in the normal-protein-intake group but significantly high in the high-protein-intake group. The results of these studies indicated that dietary diversity and appropriate protein intake (at least 1.0 g/kg/day) are effective for preventing and improving sarcopenia.

In terms of exercise, three longitudinal studies were found in a systematic review. Based on the results of a 10-year observational study targeting 3,608 Japanese individuals, the risk of developing sarcopenia decreased as the total amount of exercise and recreational physical activity and the number of steps taken increased. Meanwhile, the results of the same study indicated no significant correlation between the incidence of sarcopenia and the above activity levels and the number of steps taken in subjects aged 65 or above ^(37)^. In addition, a five-year observational study on 468 Japanese individuals aged 65–84 showed a significant correlation between the number of steps and the amount of physical activity using an accelerometer and sarcopenia onset. The relative risk of the lowest quartile of step counts for sarcopenia onset was 2.33 and 2.99 in men and women, respectively, compared with the highest quintile, and the relative risk of the lowest quartile of physical activity (three METs or higher) was 3.01 and 3.49 in men and women, respectively, compared with the highest quintile, all of which were statistically significant ^(38)^. The results of a four-year observational study in Hong Kong also showed that the risk of developing sarcopenia was significantly lower in subjects with high physical activity at the time of enrollment ^(39)^.

Not a lot of studies have been conducted for the prevention of sarcopenia in patients with morbidities. A systematic review and meta-analysis summarizing the effects of physical training in dialysis patients indicated that the grip strength and gait speed improved as a result of resistance training and aerobic exercise ^(40)^. Although most studies in this area are of a small-scale nature, exercise in addition to nutritional supplementation may help prevent sarcopenia in patients with CKD ^(41)^.

## Interventions for Sarcopenia

Diet and exercise interventions are generally well known to provide benefits for muscle strength and physical function. However, it is still unclear whether the same effect can be applied to sarcopenic older adults as defined by the EWGSOP or AWGS criteria. Thus, in the intervention section, randomized controlled trials (RCTs) targeting older adults with sarcopenia according to EWGSOP, AWGS, or others were searched ^(42)^.

However, there were no RCTs that applied inclusion criteria to older people diagnosed with sarcopenia strictly according to the EWGSOP or AWGS criteria. Therefore, RCTs including subjects diagnosed with sarcopenia according to a combination of amount of reduction in skeletal muscle mass and muscle strength/physical functions were selected. Exercise interventions administered in three RCTs comprised a comprehensive training program, including 60 min resistance exercises twice weekly for three months ^(43)^. Comparison against the control group showed that the appendicular skeletal muscle mass, usual gait speed, maximum gait speed, and knee extension muscle strength demonstrated an improvement after the training program ^(43)^. In contrast, no change in the grip strength was found. Based on these results, exercise interventions for three months or longer may help increase the skeletal muscle mass, muscle strength, and gait speed ^(43)^. The guideline committee members also showed that the evidence level was very low. Further accumulation of evidence is necessary in the future.

Nutritional interventions should offer benefits similar to those of exercise interventions for sarcopenia. In a meta-analysis that examined 12 RCTs evaluating skeletal muscle mass data, it was found that although an improvement in the physical functions was observed in three of these studies, increases in skeletal muscle mass and improvements in muscle strength were observed in one study ^(44)^. In addition, these RCTs primarily examined older people, including frail older adults. Thus, whether the conclusions reached as a result of these studies can also be extended to sarcopenic older adults is uncertain. Therefore, the guideline committee members also focused on RCTs examining sarcopenic older adults to examine the effect of nutritional intervention on sarcopenia, and they found five RCTs matching our criteria for the systematic review. The nutritional interventions included the administration of 3 g of essential amino acids twice daily, 540 mg of tea catechin supplement daily, 3 g of essential amino acids and 540 mg of tea catechin daily, and 12 g of protein and 7 g of essential amino acids daily ^(42), (43)^. As a nutritional intervention, essential amino acid supplementation was shown to be effective for improving the knee extension muscle strength. However, no significant differences were found with regard to skeletal muscle mass, fat-free mass (FFM), grip strength, knee extension muscle strength, gait speed, or Timed Up and Go performance. The results of these studies indicated that nutritional interventions for at least three months may contribute to an improvement in muscle strength. However, further studies are needed to address whether such interventions also affect skeletal muscle mass and physical functions. According to our assessment, the evidence level for nutritional interventions is very low. Further accumulation of evidence is necessary to clarify these issues.

As a result, a systematic review in which a meta-analysis of four articles was performed to verify the effects of combined interventions in three studies was conducted. In addition, Zdzieblik et al. investigated the effect of 60 min resistance training using exercise machines three times a week while receiving 15 g of collagen peptide or placebo for three months ^(45)^. A subgroup meta-analysis incorporating these four RCTs was conducted to compare the effects of exercise interventions alone versus a combination of nutritional and exercise interventions, as well as a combined exercise and nutritional intervention versus a nutritional intervention alone. Although combined exercise and nutritional interventions tended to increase FFM after three months in the four RCTs, no significant changes in the appendicular skeletal muscle mass, grip strength, knee extension muscle strength, or normal/maximum gait speed were observed. The guideline committee members also analyzed three RCTs and found that combined exercise and nutritional interventions were effective in improving the knee extension muscle strength after three months. However, no significant changes were observed with respect to the appendicular skeletal muscle mass, grip strength, or usual/maximum gait speed. Although the additive effects of exercise and nutritional interventions could not be demonstrated through this systematic review, Rondanelli et al. reported increased FFM and improved muscle strength in older adults with low skeletal muscle mass who were supplemented with whey protein, essential amino acids, and vitamin D for 12 weeks after all the subjects had completed an exercise intervention ^(46)^. Accordingly, a combination of exercise and nutrition should be provided as an effective therapeutic intervention for sarcopenia.

Evidence regarding the efficacy of drug therapy for sarcopenia is currently inadequate. Only one article concerning the verification of the efficacy of drug therapy as a treatment for sarcopenia in older adults was found. Based on the results, although increased skeletal muscle mass was observed as a therapeutic effect of drugs administered to older people with sarcopenia, no increased muscle strength or gait speed was clearly shown. No report showed the therapeutic effects of drugs in men with sarcopenia. Large-scale interventions of various subject populations are needed in the future.

In conclusion, sarcopenia is considered to be a significant threat for superaged societies. It is critical for all healthcare professionals to understand what sarcopenia is and how we can diagnose, prevent, and treat it. I hope that these clinical sarcopenia guidelines will accelerate the research on sarcopenia in the future. Based on the evidence, these guidelines will be revised several years later.

## Article Information

### Conflicts of Interest

None

### Acknowledgement

This is an executive summary of the clinical guidelines of sarcopenia that were published in Geriatr Gerontol Int. 2018;18 Suppl 1:1-44. The original version is available at https://onlinelibrary.wiley.com/toc/14470594/2018/18/S1. The Editors-in-Chief of Geriatrics & Gerontology International and JMA Journal and the publisher of the original version have permitted the publication of this manuscript. I would like to thank all the committee members for their dedicated work to the development of the guidelines.
